# Supplementary material for: Heat adaptation of phage T7 under an extended genetic code
Source: Virus Evol. 2021 Dec 1;7(2):veab100. doi: 10.1093/ve/veab100 (PMC8923235; doi:10.1093/ve/veab100)
Supplement: veab100_Supp [file veab100_supp.zip › Sequencing_Analysis.zip!/Sequencing_Analysis/S3/evidence/JC_SIDE_2_46.html]

BRESEQ :: Evidence


| Predicted mutation | | | | | | |
| --- | --- | --- | --- | --- | --- | --- |
| evidence | seq id | position | mutation | annotation | gene | description |
| MC JC | NC\_001604.1 | 1,257 | Δ1,480 bp | intergenic (–/–) | – / – | –/– |

| Missing coverage evidence... | | | | | | | | | | |
| --- | --- | --- | --- | --- | --- | --- | --- | --- | --- | --- |
|  |  |  | seq id | start | end | size | ←reads | reads→ | gene | description |
| \* | \* | ÷ | NC\_001604.1 | 1257 | 2736 | 1480 | 1916 [0] | [1] 1913 | *–/–* | –/– |

| New junction evidence | | | | | | | | | | | |
| --- | --- | --- | --- | --- | --- | --- | --- | --- | --- | --- | --- |
|  | | seq id | position | reads (cov) | reads (cov) | score | skew | freq | annotation | gene | product |
| \* | ? | NC\_001604.1 | = 1256 | 0 (0.000) | 1845 (1.120) | 330/574 | 0.0 | 100% | intergenic (–/–) | *–/–* | –/– |
|  | ? | NC\_001604.1 | 2737 = | 0 (0.000) |  |  |  |  | intergenic (–/–) | *–/–* | –/– |

Only 100 of 1913 total aligned reads displayed.  

`GGATTGCGAGCGTTTCAACAATGATGCCCATTATAAATACGCTGAGATTGCAAGCGACATCATTGATTGCAATTCGGATGAGCATGATGAGTTAACTGGATGGGATGGTGAGTTTGTTGAAACTTGTAAACTAATCCGCAAGTTCTTTGAGGGCATCGCCTCATTCGACATGCATAGCGGGAACATCATGTTCTCAAATGGAGACGTACCATACATCACCGACCCGGTATCATTCTCGCAGAAGAAAGACGGTGGCGCATTCAGCATCGACCCTGAGGAACTCATCAAGGAAGTCGAGGAAGTCGCACGACAGAAAGAAATTGACCGCGCTAAGGCCCGTAAAGAACGTCACGAGGGGCGCTTAGAGGCACGCAGATTCAAACGTCGCAACCGCAAGGCACGTAAAGCACACAAAGCTAAGCGCGAAAGAATGCTTGCTGCGTGGCGATGGGCTGAACGTCAAGAACGGCGTAACCATGAGGTAGCTGTAGATGTACTAGGAAGAACCAATAACGCTATGCTCTGGGTCAACATGTTCTCTGGGGACTTTAAGGCGCTTGAGGAACGAATCGCGCTGCACTGGCGTAATGCTGACCGGATG  >  NC_001604.1/2437‑3037`  

                                                                                                                                                                                                                                                                                                            `|`                                                                                                                                                                                                                                                                                                              
`tttcgaccacgcttacgaaatgctgaaagaaaacatccgttatgatgacatccgtgacactgatgacctgcacgatgctattcacatggctgccgataatgcagtttcgcactactacgctgacatctttagcgtaatggcaagtgagggcattgaccttgagttcgaagactctggtctgatgcctgacaccaaggacgtaatccgcatcctgcaagcgcgtatctatgagcaattaacgattgacctctgggaagacgcagaagacttgctcaatgaatacttggaggaagtcgaggaa                                                                                                                                                                                                                                                                                                              <  1:242969‑M2/1‑1 (MQ=255)`  
         `cgcttacgaaatgctgaaagaaaacatctgttatgatgacatccgtgacactgatgacctgcacgatgctattcacatggctgccgataatgcagttccgcactactacgctgacatctttagcgtaatggcaagtgagggcattgaccttgagttcgaagactctggtctgctgcctgacaccaatgacgtaatccgcatcctgcaagcgcgtatctatgagcaattaacgattgacctctgggaagacgcatatgactttctcaatgaatccttggaggaagtcgaggaAGTCGCACGa                                                                                                                                                                                                                                                                                                     >  1:164363‑M2/292‑301 (MQ=255)`  
          `gcttacgaaatgatgaaagaaaacatccgttatgatgacatccgtgacactgatgacctgcacgatgctattcacatggctgccgataatgcagttccgcactactacgctgacatctttagcgtaatggcaagtgagggcattgaccttgagttcgaagactctggtctgatgcctgacaccaaggacgtaatccgcatcctgcaagcgcgtatctatgagcaattaacgattgacctctgggaagacgcagaagacttgctcaatgaatacttggaggaagtcgaggaAGTCGCACGAc                                                                                                                                                                                                                                                                                                    <  1:252179‑M2/11‑1 (MQ=255)`  
             `tacgaaatgctgaaagaaaacatccgttatgatgacatccgtgacactgatgacctgcacgatgctattcacatggctgccgataatgcagttccgcactactacgctgacatctttagcgtaatggcaagtgagggcattgaccttgagttcgaagactctggtctgatgcctgacaccaaggacgtaatccgcatcctgcaagcgcgtatctatgagcaattaacgattgacctctgggaagacgcagaagacttgctcaatgaatacttggaggaagtcgaggaAGTCGCACGACaga                                                                                                                                                                                                                                                                                                 <  1:210112‑M2/14‑1 (MQ=255)`  
                    `tgctgaaagaaaacatccgttatgatgacatccgtgacactgatgacctgcacgatgctattcacatggctgccgataatgcagttccgcactactacgctgacatctttagcgtaatggcaagtgagggcattgaccttgagttcgaagactctggtctgatgcctgacaccaaggacgtaatccgcatcctgcaagcgcgtatctatgagcaattaacgattgacctctgggaagacgcagaagacttgctcaatgaatacttggaggaagtcgaggaAGTCGCACGACAGAAAGAAAt                                                                                                                                                                                                                                                                                          <  1:145017‑M2/21‑1 (MQ=255)`  
                    `acaggaaagaaaacatccgttatgatgacatccgtgacactgatgacctgcacgatgctattcacatggctgccgataatgcagttccgcactactacgctgacatctttagcgtaatggcaagtgagggcattgaccttgagttcgaagactctggtctgatgcctgacaccaaggacgtaatccgcatcctgcaagcgcgtatctatgagcaattaacggttgacctctgggaagacgcagaagacttgctcaatgaatacttggaggaagtcgaggaAGTCGCACGACAGAAAGAAAt                                                                                                                                                                                                                                                                                          <  1:285879‑M2/21‑1 (MQ=255)`  
                                  `atccgttatgatgacatccgtgacactgatggcctgaacgatgctattcacatggctgacgatgatgcagttccgcactactacgctgacatctttagcgtaatggcaagtgagggcattgaccttgagttcgaagactctggtctgatgcctgacaccaaggacgtaatccgcatcctgcaagcgcgtatctatgagcaattaacgattgacctctgggaagacgcagaagacttgctcaatgaatacttggaggaagtcgaggaAGTCGCACGACAGAAAGAAATTGACCGCGCTAAgg                                                                                                                                                                                                                                                                            <  1:182433‑M2/35‑1 (MQ=255)`  
                                   `cccgttatgatgacatccgtgacactgaggacctgctcgatgctattcacatggcggccgataatgcagttccgcactactacgctgacatcattagcgtaatggcaagtgagggcattgaccttgagttcgaagactctggtctgatgcctgacactaaggacgtaatccgcatcctgcaagcgtgtatctatgagcaattaacgattgacctctgggaagacgcagaagacttgctcaatgaatacttggaggaagtcgaggaAGTCGCACGACAGAAAGAAATTGACCGCGCTAAGGc                                                                                                                                                                                                                                                                           <  1:323879‑M2/36‑1 (MQ=255)`  
                                         `atgatgacatccgtgacactgatgacctgcacgatgctattcacatggctgccgataatgcagttccgcactactacgctgacatctttagcgtaatggcaagtgagggcattgaccttgagttcgaagactctggtctgatgcctgacaccaaggacgtaatccgcatcctgcaagcgcgtatctatgagcaattaacgattgacctctgggaagacgcagaagacttgctcaatgaatacttggaggaagtcgaggaAGTCGCACGACAGAAAGAAATTGACCGCGCTAAGGCCCGTaa                                                                                                                                                                                                                                                                     <  1:124728‑M2/42‑1 (MQ=255)`  
                                         `atgatgacatccgtgacaatgatgacctgcacgatgctattaaaatggctgccgataatgcagttccgcactactacgctgacatctttagcgtaatggcaaatgagggcattgaccttgagttcgaagactctggtctgatgcatgacaccaaggacgtaattcgcatcctgcaagcgcgtatctatgagcaattaacgattgacctctgggaagacgcagaagacttgctcaatgaatacttggaggaagtcgaggaAGTCGCACGACAGAAAGAAATTGACCGCGCTAAGGCCCGTaa                                                                                                                                                                                                                                                                     <  1:142398‑M2/42‑1 (MQ=255)`  
                                           `gatgacggccgtgacacggatgacccgcacgatgctattcacatggctgccgatgatgcagttccgcactacgacggtgacatcgttagcggaatggcaagtgagggcattgaccttgagttcgaagactctggtctgatgcctgacaccaaggacgtaaaccgcatcctgcaagcgcgtatctatgagcaagtaacgattgacctctgggaagacgcagaagacttgctaaatgaatacttggaggaagtcgaggaAGTCGCACGACAGAAAGAAATTGACCGCGCTAAGGCCCGtaaaa                                                                                                                                                                                                                                                                   <  1:1609‑M2/44‑2 (MQ=255)`  
                                           `atccgttatgatgacatccgtgacactgatgacctgcacgatgctattcacatggctgccgataatgcagttccgcactactacgctgacatctttagcgtaatggcaagtgagggcattgaccttgagttcgaagactctggtctgatgcctgacaccaaggacgtaatccgcatcctgcaagcgcgtatctatgagcaattaacgattgacctctgggaagacgcagaagacttgctcaatgaatacttggaggaAGTCGCACGACAGAAAGAAATTGACCGCGCTAAGGCCCGTAAAg                                                                                                                                                                                                                                                                   <  1:294430‑M2/44‑1 (MQ=255)`  
                                                   `ccgtgacactgatgacctgcacgatgctattcacatggctgccgataatgcagttccgcactactacgctgacatctttagcgtaatggcaagtgagggcattgaccttgagttcgaagactctggtctgatgcctggcaccaaggacgtaatccgcatcctgcaagcgcgtatctatgagcaattaacgattgacctctgggaagacgcagaagacttgctcaatgaatacttggaggaagtcgaggaAGTCGCACGACAGAAAGAAATTGACCGCGCTAAGGCCCGTAAAGAACGTCAc                                                                                                                                                                                                                                                           >  1:148419‑M2/250‑301 (MQ=255)`  
                                                   `ccgtgacactgatgacctgcacgatgctattcacatggctgccgataatgcagttccgcactactacgctgacatctttagcgtaatggcaagtgagggcattgaccttgagttcgaagactctggtctgatgcctgacaccaaggacgtaatccgcatcctgcaagcgtgtctctatgagcaattaacgattgacctctgggaagacgcagaagacttgctcaatgaatacttggaggaagtcgaggaAGTCGCACGACAGAAAGAAATTGACCGAGCtaaggcccgtaactgtctctta                                                                                                                                                                                                                                                           >  1:97393‑M2/250‑291 (MQ=255)`  
                                                    `cgtgacactgatgacctgcacgatgctattcacatggctgccgataatgcagttccgcactactacgctgtcatctttagcgtaatggcaagtgagggcattgaccttgagttcgaagactctggtctgatgcctgacaccaaggacgtaatccgcatcctgcaagcgcgtatctatgagcaattaacgattgacctctgggaagacgcagaagacttgctcaatgaatacttggaggaagtcgaggaAGTCGCACGACAGAAAGAAATTGACCGCGCTAAGGCCCGTAAAGAACGTCACg                                                                                                                                                                                                                                                          >  1:13981‑M2/249‑301 (MQ=255)`  
                                                         `acactgatgacctgcacgatgctattcacatggctgccgataatgcagttccgcactactacgctgacatctttagcgtaatggcaagtgagggcattgaccttgagttcgaagactctggtctgatgcctgacaccaaggacgtaatcccatcctgcaagcgcgtatctatgagcaattaacgattgacctctgggaagacgcagaagacttgctcaatgaatacttggaggaagtcgaggaAGTCGCACGACAGAAAGAAATTGACCGCGCTAAGGCCCGTAAAGAACGTCACGAgggg                                                                                                                                                                                                                                                     >  1:51284‑M2/244‑301 (MQ=255)`  
                                                                          `atgctattcacatggctgccgataatgcagttccgcactactacgctgacatctttagcgtaatggcaagtgagggcattgaccttgagttcgaagactctggtctgatgcctgacaccaaggacgtaatccgcatcctgcaagcgcgtatctatgagcaattaacgattgacctctgggaagacgcagaagacttgctcaatgaatacttggaggaagtcgaggaAGTCGCACGACAGAAAGAAATTGACCGCGCTAAGGCCCGTAAAGAACGTCACGaggggcgcttagctgtctcttt                                                                                                                                                                                                                                    >  1:196887‑M2/227‑291 (MQ=255)`  
                                                                          `atgctattcacatggctgccgataatgcagttccgcactactacgctgacatctttagcgtaatggcaagtgagggcattgaccttgagttcgaagactctggtctgatgcctgacaccaaggacgtaatccgcatcctgcaagcgcgtatctatgagcaattaacgattgacctctgggaagacgcagaagacttgctcaatgaatacttggaggaagtcgaggaAGTCGCACGACAGAAAGAAATTGACCGCGCTAAGGCCCGTAAAGAACGTCACGAAGGGCGCTTAGAGGCACGCAg                                                                                                                                                                                                                                    >  1:332776‑M2/227‑301 (MQ=255)`  
                                                                               `attcacatggctgccgataatgcagttccgcactactacgctgacatctttagcgtaatggcaagtgagggcattgaccttgagttcgaagactctggtctgatgcctgacaccaaggacgtaatccgcatcctgcaagcgcgtatctatgagcaattaacgattgacctctgggaagacgcagaagacttgctcaatgaatacttggaggaagtcgaggaAGTCGCACGACAGAAAGAAATTGACCGCGCTAAGGCCCGTAAAGAACGTCACGAGGGGCGCTTAGAGGCACGCAGATTCa                                                                                                                                                                                                                               >  1:275919‑M2/222‑301 (MQ=255)`  
                                                                                    `catggctgccgataatgcagttccgcactactacgctgacatctttagcgtaatggcaagtgagggcattgaccttgagttcgaagactctggtctgatgcctgacaccaaggacgtaatccgcatcctgcaagcgcgtatctatgagcaattaacgattgacctctgggaagacgcagaagacttgctcaatgaatacttggaggaagtcgaggaAGTCGCACGACAGAAAGAAATTGACCGCGCTAAGGCCCGTAAAGAACGTCACGAGGGGCGCTTAGAGGCACGCAGATTCAAACGt                                                                                                                                                                                                                          <  1:313982‑M2/85‑1 (MQ=255)`  
                                                                                             `cgataatgcagttccgcactactacgctgacatctttagcgtaatggcaagtgagggcattgaccttgagttcgaagactctggtctgatgcctgacaccaaggacgtaatccgcatcctgcaagcgcgtatctatgagcaattaacgattgacctctgggaagacgcagaagacttgctcaatgaatacttggaggaagtcgaggaAGTCGCACGACAGAAAGAAATTGACCGCGCTAAGGCCCGTAAAGAACGTCACGAGGGGCGCTTAGAGGCACGCAGATTCAAACGTCGCAACCGc                                                                                                                                                                                                                 >  1:132022‑M2/208‑301 (MQ=255)`  
                                                                                              `cgataatgcagttccgcactactacgctgacatctttagcgtatggcaagtgagggcattgaccttgagttcgaagactctggtctgatgcctgacaccaaggacgtaatccgcatcctgcaagcgcgtatctatgagcaattaacgattgacctctgggaagacgcagaagacttgctcaatgaatacttggaggaagtcgaggaAGTCGCACGCCAGAAAGAAATTGACAGCGCTAAGGCCCGTAAAGAACATCACGAGGGGCGCGTAGAGGCACGCAGATTCAAACGTCGCAACCCCa                                                                                                                                                                                                                >  1:265677‑M2/207‑301 (MQ=255)`  
                                                                                                  `atgcagttccgcactactacgctgacatctttagcgtaatggcaagtgagggcattgaccttgagttcgaagactctggtctgatgcctgacaccaaggacgtaatccgcatcctgcaagcgcgtatctatgagcaattaacgattgacctctgggaagacgcagaagacttgctcaatgaatacttggatgaagtcgaggaAGTCGCACGACAGAAAGAAATTGACCGCGCTAAGGCCCGTAAAGAACGTCACGAGGGGCGCTTAGAGGCACGCAGATTCAAACGTCGCAACCGCAAGGc                                                                                                                                                                                                            <  1:302014‑M2/99‑1 (MQ=255)`  
                                                                                                           `ggaagggtataagagacatcttgagcgtaatggcaagtgagggcattgaccttgagttcgaagactctggtctgatgcctgacaccaaggacgtaatccgcatcctgcaagcgcgtatctatgagcaattaacgattgacctctgggaagacgcagaagacttgctcaattagtacttggaggaagtcgaggaAGTCGCACGACAGAAAGAAATTGACCGCGCTAAGGCCCGTAAAGAACGTCACGAGGGGCGCTTAGAGGCACGCAGATTCAAACGTCGCAACCGCAAGGCACGTAAAGc                                                                                                                                                                                                   <  1:75359‑M2/108‑1 (MQ=255)`  
                                                                                                           `atgcagttccgcactactacgctgacatctttagcgtaatggcaagtgagggcattgaccttgagttcgaagactctggtctgatgcctgacaccaaggacgtaatccgcatcctgcaagcgcgtatctatgagcaattaacgattgacctctgggaagacgcagaagacttgctcaatgaatacttggaggaAGTCGCACGACAGAAAGAAATTGACCGCGCTAAGGCCCGTAAAGAACGTCACGAGGGGCGCTTAGAGGCACGCAGATTCAAACGTCGCAACCGCAAGGCACGTAAAGc                                                                                                                                                                                                   >  1:207759‑M2/194‑301 (MQ=255)`  
                                                                                                               `ctactacgctgacatctttagcgtaatggcaagtgagggcattgaccttgagttcgaagactctggtctgatgcctgacaccaaggacgtaatccgcatcctgcaagcgcgtatctatgagcaattaacgattgacctctgggaagacgcagaagacttgctcaatgaatacttggaggaagtcgaggcAGTCGCACGACAGAAAGAAATTGACCGCGCTAAGGCCCGTAAAGAACGTCACGAGGGGCGCTTAGAGGCACTCAGATTCAAACGTCGCCACCGCAAGGCACGTAAAGcacac                                                                                                                                                                                               >  1:231055‑M2/190‑301 (MQ=255)`  
                                                                                                                 `actacgctgacatctttagcgtaatggcaagtgagggcattgaccttgagttcgaagactctggtctgatgcctgacaccaaggacgtaatccgcatcctgcaagcgcgtatctatgagcaattaacgattgacctctgggaagacgcagaagacttgctcaatgaatacttggaagaagtcgaggaAGTCGCACGACAGAAAGAAATTGACCGCGCTAAGGCCCGTAAAGAACGTCACGAGGGGCGCTTAGAGGCACGCAGATTCAAACGTCGCAACCGCAAGGCACGTAAAGCACACaa                                                                                                                                                                                             >  1:308406‑M2/188‑301 (MQ=255)`  
                                                                                                                  `ctacgatgacatctttagcgtaatggcaagtgagggcattgaccttgagttcgaagactctggtctgatgcctgacaccaaggacgtaatccgcatcctgcaagcgcgtatctatgagcaattaacgattgacctctgggaagacgcagaagacttgctcaatgaatacgtggaggaagtcgaggaAGTCGCACGACAGAAAGAAATTGACCGCGCTAAGGCCCGTAAAGAACGTCACGAGGGGCGCTTAGAGGCACGCAGATTCAAACGTCGCAACCGCAAGGCACGTAAAGCACACaaa                                                                                                                                                                                            <  1:177446‑M2/115‑1 (MQ=255)`  
                                                                                                                    `acgctgacatctttagcgtaatggcaagtgagggcattgaccttgagttcgaagactctggtctgatgcctgacaccaaggacgtaatccgcatcctgcaagcgcgtatctatgagcaattaacgattgacctctgggaagacgcagaagacttgctcaatgaatacttggaggaagtcgaggaAGTCGCACGACAGAAAGAAATTGACCGCGCTAAGGCCCGTAAAGAACGTCACGAGGGGCGCTTAGAGGCACGCAGATTCAAACGTCGCAACCGCAAGGCACGTAAAGCACACAAAGc                                                                                                                                                                                          <  1:216544‑M2/117‑1 (MQ=255)`  
                                                                                                                         `gacatctttagcgtaatggcaagtgagggcattgaccttgagttcgaagactctggtctgatgcctgacaccaaggacgtaatccgcatcctgcaagcgcgtatctatgagcaattaacgattgacctctgggaagacgcagaagacttgctcaatgaatacttggaggaagtcgaggaAGTCGCACGACAGAAAGAAATTGACCGCGCTAAGGCCCGTAAAGAACGTCCCGAGGGGCGCTTAGAGGCACGCAGATTCAAACGTCGCAACCGCAAGGCACGTAAAGCACACAaagctctgt                                                                                                                                                                                     >  1:194179‑M2/180‑297 (MQ=255)`  
                                                                                                                              `ctttagcgtaatggcaagtgagggcattgaccttgagttcgaagactctggtctgatgcctgacaccaaggacgtaatccgcatcctgcaagcgcgtatctatgagcaattaacgattgacctctgggaagacgcagaagacttgctcaatgaatacttggaggaagtcgaggaAGTCGCACGACAGAAAGAAATTGACCGCGCTAAGGCCCGTAAAGAACGTCACGAGGGGCGCTTAGAGGCACGCAGATTCAAACGTCGCAACCGCAAGGCACGTAAAGCACACAAAGCTAAGCGCGaa                                                                                                                                                                                >  1:262038‑M2/175‑301 (MQ=255)`  
                                                                                                                                   `gcgtaatggcaagtgagggcattgaccttgagttcgaagactctggtctgatgcctgacaccaaggacgtaatccgcatcctgcaagcgcgtatctatgagcaattaacgattgacctctgggaagacgcagaagacttgctcaatgaatacttggaggaagtcgaggaAGTCGCACGACAGAAAGAAATTGACCGCGCTAAGGCCCGTAAAGAACGTCACGAGGGGCGCTTAGAGGCACGCAGATTCAAACGTCGCAACCGCAAGGCACGTAAAGCACACAAAGCTAAGCGCGAAAGAAt                                                                                                                                                                           >  1:110567‑M2/170‑301 (MQ=255)`  
                                                                                                                                     `gtaatggcaagtgagggcattgaccttgagtgcgaagactctggtctgatgcctgacaccaaggacgtaatccgcatcctgcaagcgcgtatctatgagcaattaacgattgacctctgggaagacgcagaagacttgctcaatgaatacttggaggaagtcgaggaAGTCGCACGACAGAAAGAAATTGACCGCGCTAAGGCCCGTAAAGAACGTCACGAGGGGCGCTTAGAGGCACGCAGATTCAAACGTCGCAACCGCAAGGCACGTAAAGCACACAAAGCTAAGCGCGAAAGAAtgc                                                                                                                                                                         <  1:20459‑M2/134‑1 (MQ=255)`  
                                                                                                                                        `atggcaagtgagggcattgaccttgagttcgaagactctggtctgatgcctgacaccaaggacgtaatccgcatcctgcaagcgcgtatctatgagcaataaacgattgacctctgggaagacgcagaagacttgctcaatgaatacttggaggaagtcgaggaAGTCGCACGACAGAAAGAAATTGACCGCGCTAAGGCCCGTAAAGAACGTCACGAGGGGCGCTTAGAGGCACGCAGATTCAAACGTCGCAACCGCAAGGCACGTAAAGCACACAAAGCTAAGCGCGAAAGAAtgcttg                                                                                                                                                                      >  1:95406‑M2/165‑301 (MQ=255)`  
                                                                                                                                         `tggcaagtgagggcattgaccttgagttcgaagactctggtctgatgcctgacaccaaggacgtaatccgcatcctgcaagcgcgtatctatgagcaattaacgattgacctctgggaagacgcagaagacttgctcaatgaatacttggaggaagtcgaggaAGTCGCACGACAGAAAGAAATTGACCGCGCTAAGGCCCGTAAAGAACGTCACGAGGGGCGCTTAGAGGCACGCAGATTCAAACGTCGCAACCGCAAGGCACGTAAAGCACACAAAGCTAAGCGCGAAAGAAtgcttgc                                                                                                                                                                     >  1:254428‑M2/164‑301 (MQ=255)`  
                                                                                                                                               `gtgagggcattgaccttgagttcgaagactctggtctgatgcctgacaccaaggacgtaatccgcatcctgcaagcgcgtatctatgagcaattaacgattgacctctgggaagacgcagaagacttgctcaatgaatacttggaggaagtcgaggaAGTCGCACGACAGAAAGAAATTGACCGCGCTAAGGCCCGTAAAGAACGTCACGAGGGGCGCTTAGAGGCACGCAGATTCAAACGTCGCAACCGCAAGGCACGTAAAGCACACAAAGCTAAGCGCGAAAGAATGCTTGCTGCGTg                                                                                                                                                               >  1:269718‑M2/158‑301 (MQ=255)`  
                                                                                                                                                `tgagggcattgaccttgagttcgaagactctggtctgatgcctgacaccaaggacgtaatccgcatcctgcaagcgcgtatctatgagcaattaacgattgacctctgggaagacacagaagacttgctcaatgaatactttgaggaagtcgaggaAGTCGCACGACAGAAAGAAATTGACCGCGCTAAGGCCCGTAAAGAACGTCACGAGGGTCGCTTAGAGGCACGCAGATTCAAACGTCGCAACCGCAAGGCACGTAAAGCACACAAAGCCAAGCGCGAAAGAATGCTTGCTGCGTgg                                                                                                                                                              >  1:88883‑M2/157‑301 (MQ=255)`  
                                                                                                                                                        `ttgaccttgagttcgaagactctggtctgatgcctgacaccaaggacgtaatccgcatcctgcaagcgcgtatctatgagcaattaacgattgacctctgggaagacgcagaagacttgctcaatgaatacatggaggaagtcgaggaAGTCGAACGACAGAAAGAAATTGACCGCGCTAAGGCCCGTAAAGAACGTCACGAGGGGCGCTTAGAGGCACGCAGATTCAAACGTCGCAACCGCAAGGCACGTAAAGCACACAAAGCTAAGCGCGAAAGAATGCTTGCTGCGTGGCGATGGGc                                                                                                                                                      <  1:103597‑M2/153‑1 (MQ=255)`  
                                                                                                                                                          `gaccttgagttcgaagactctggtctgatgcctgacaccaaggacgtaatccgcatcctgcaagcgcgtatctatgagcaattaacgattgacctctgggaagacgcagaagacttgctcaatgaatacttggaggaagtcgaggaAGTCGCACGACAGAAAGAAATTGACCGCGCTAAGGCCCGTAAAGAACGTCACGAGGGGCGCTTAGAGGCACGCAGATTCAAACGTCGCAACCGCAAGGCACGTAAAGCACACAAAGCTAAGCGCGAAAGAATGCTTGCTGCGTGGCGAGGGGCTg                                                                                                                                                    >  1:237047‑M2/147‑301 (MQ=255)`  
                                                                                                                                                           `accttgagttcgaagactctggtctgatgcctgacaccaaggacgtaatccgcatcctgcaagcgcgtatctatgagcaattaacgattgacctctgggaagacgcagaagacttgctcaatgaatacttggaggaagtcgaggaAGTCGCACGACAGAAAGAAATTGACCGCGCTAAGGCCCGTAAAGAACGTCACGAGGGGCGCTTAGAGGCACGCAGATTCAAACGTCGCAACCGCAAGGCACGTAAAGCACACAAAGCTAAGCGCGAAAGAATGCTTGCTGCGTGGCGATGGGCTGa                                                                                                                                                   >  1:227781‑M2/146‑301 (MQ=255)`  
                                                                                                                                                             `cttgagttcgaagactctggtctgatgcctgacaccaaggacgtaatccgcatcctgcaagcgcgtatctatgagcaattaacgattgacctctgggaagacgcagaagacttgctcaatgaatacttggaggaagtcgaggaAGTCGCACGACAGAAAGAAATTGACCGCGCTAAGGCCCGTAAAGAACGTCACGAGGGGCGCTTAGAGGCACGCAGATTCAAACGTCGCAACCGCAAGGCACGTAAAGCACACAAAGCTAAGCGCGAAAGAATGCTTGCTGCGTGGCGATGGGCTGAAc                                                                                                                                                 <  1:282627‑M2/158‑1 (MQ=255)`  
                                                                                                                                                                 `agttcgaagactctggtctgatgcctgacaccaaggacgtaatccgcatcctgcaagcgcgtatctatgagcaattaacgattgacctctgggaagacgcagaagacttgctcaatgaatacttggaggaagtcgaggaAGTCGCACGACAGAAAGAAATTGACCGCGCTAAGGCCCGTAAAGAACGTCACGAGGGGCGCTTAGAGGCACGCAGATTCAAACGTCGCAACCGCAAGGCACGTAAAGCACACAAAGCTAAGCGCCAAATAATGCTTGCTGCGTggcgatgggccagaagtca                                                                                                                                             >  1:249725‑M2/140‑292 (MQ=255)`  
                                                                                                                                                                     `cgaagaatctggtctgatgcatgacaccaaggacgtaatccgcatcctgcaagcgcgtatgtatgagcaattaacgattgacctctgggaagacgcagaagacttggtcaatgaatacttggaggaagtcgaggaAGTCGCACGACAGAAAGAAATTGACCGCGCTAAGGCCCTTAAAGAACGTCACGAGGGGCGCTTAGAGGCACGCAGATTCAAACGTCGCAACCGCAAGGCACGTAAAGCACACAAAGCTAAGCGCGAAAGAATGCTTGCTGCGTGGCGATGGGCTGAACGTCAAGaa                                                                                                                                         <  1:245896‑M2/166‑1 (MQ=255)`  
                                                                                                                                                                      `gaagactctggtctgatgcctgacaccaaggacgtaatacgcatcctgcaagcgagtatctatgagcaattaacgatgaacctcggggaagacgaagaagacttgctcaatgaatacttgggggaagtcgaggaGGTCACGCGAAAGAAAGAAATTGACCGCGCTAAGGCCCGTAAAGAACGTCGCGAGGGGAGCTTAGAGGCACGCAGATTCAAACGTCGCAACCGCAAGGCACGTAAAGCACACAAAGCTAAGCGCGAAAGAATGCTTGCTGCGTGGCGATGGGCTGAACGTCAAGAAc                                                                                                                                        <  1:199186‑M2/167‑1 (MQ=255)`  
                                                                                                                                                                      `gaagactctgggctgattcctgacacgaaggacgtaatccgcatcctgcaagagggtatcgatgagcaattaacgattgacctctgggaagacacagaagacttgcacaatgaatacttggaggaagtcgaggaAGTCGCACGACAGAAAGAAATTGACCGCGCTAAGGCCCGTAAAGAACGTCACGAGGGGCGCTTAGAGGCACGCAGATTCAAACGTCGCACCCGCAAGGCACGTAAAGCACACAAAGCTAAGCGCGAAAGAATGCTTGCTGCGTGGCGATGGGCTGAACGTCAAGAAc                                                                                                                                        <  1:347148‑M2/167‑1 (MQ=255)`  
                                                                                                                                                                           `ctctggtctgatgcctgacaccaaggacgtaatccgcatcctgcaagcgtgtatctatgagcaattaacgattgacctctgggaagacgcagaagacttgctcaatgaatacttggaggaagtcgaggaAGTCGCACGACAGAAAGAAATTGACCGCGCTAAGGCCCGTAAAGAACGTCACGAGGGGCGCTTAGAGGCACGCAGATTCAAACGTCGCAACCGCAAGGCACGTAAAGCACACAAAGCTAAGCGCGAAAGAATGCTTGCTGCGTGGcgatgggctgaacgctgtctcttatac                                                                                                                                   >  1:298608‑M2/130‑288 (MQ=255)`  
                                                                                                                                                                           `ctctggtctgatgcctgacaccaaggacgtaatccgcatcctgcaagcgcgtatctatgagcaattaacgattgacctctgggaagacgcagaagacttgctcaatgaatacttggaggaagtcgaggaAGTCGCACGACAGAAAGAAATTGACCGCGCTAAGGCCCGTAAAGAACGTCACGAGGGGCGCTTAGAGGCACGCAGATTCAAACGTCGCAACCGCAAGGCCCGTAAAGCACACAAAGCTAAGCGCGAAAGAATGCTTGCTGCGTGGAGATGGGCGGAACGGCAAGAACGGCGt                                                                                                                                   >  1:330844‑M2/130‑301 (MQ=255)`  
                                                                                                                                                                              `gggtttgatggcggacaacaaggacgtaatccgcatcctgcaagcacgtatctatgagcaattaacgattgacctgtgggaagaagcaaaagacttgctcaatgaatacttggagaaagtcgaggaAGTCGCACGAAAGAAAGAAATTGACCGCGCTAAGGCACGTAAAGAACGTCACGAGGGGCGCTTAGAGGCACGCAGATTCAAACGTCGAAACCGCAAGGCACGTAAAGCACACAAAGCTAAGCGCGAAAGAATGCTTGCTGCGTGGCGATGGGCTGAACGTCAAGAACGGCGTAAc                                                                                                                                <  1:238884‑M2/175‑1 (MQ=255)`  
                                                                                                                                                                               `ggtctgatgcctgacaccaaggacgtaatccgcatcctgcaagcgcgtatctatgagcaattaacgattgacctctgggaagacgcagaagacttgctcaatgaatacttggaggaagtcgaggcAGTCGCACGACAGAAAGAAATTGACCGCGCTAAGGCCCGTAAAGAACGTCACGAGGGGCGCTTAGAGGCACGCAGATTCAAACGTCGCAACCGCAAGGCACGTAAAGCACACAAAGCTAAGCGCGAAAGAATGCTTGCTGCGTGGCGATGGGCTGAACGTCAAGAACGGCGTAAcc                                                                                                                               >  1:316256‑M2/126‑301 (MQ=255)`  
                                                                                                                                                                               `ggtctgatgcctgacaccaaggacgtaatccgcatcctgcaagcgcgtatctatgagcaattaacgattgacctctgggaagacgcagaagacttgctcaatgaatacttggaggaagtcgaggaAGTCGCACGACAGAAAGAAATTGACCGCGCTAAGGCCCGTAAAGAACGTCACGAGGGGCGCTTAGAGGCACGCAGATTCAAACGTCGCAACCGCAAGGCACGTAAAGCACACAAAGCTAAGCGCGAAAGAATGCTTGCTGCGTGGCGATGGGCTGAACGTCAAGAACGGCGTAAcc                                                                                                                               >  1:106397‑M2/126‑301 (MQ=255)`  
                                                                                                                                                                               `ggtctgatgcctgacaccaaggacgtaatccgcatcctgcaagcgcgtatctatgagcaattaacgattgacctctgggaagacgcagaagacttgctcaatgaatacttggaggaagtcgaggaAGTCGCACGACAGAAAGAAATTGACCGCGCTAAGGCCCGTAAAGAACGTCACGAGGGGCGCTTAGAGGCACGCAGATTCAAACGTCGCAACCGCAAGGCACGTAAAGCACACAAAGCTAAGCGCGAAAGAATGCTTGCTGCGTGGCGATGGGCTGAACGTCAAGAACGGCGTAAcc                                                                                                                               >  1:310424‑M2/126‑301 (MQ=255)`  
                                                                                                                                                                               `ggtctgatgcctgacaccaaggacgtaatccgcatcctgcaagcgcgtatctatgagcaattaacgattgacctctgggaagacgcagaagacttgctcaatgaatacttggaggaagtcgaggaAGTCGCACGACAGAAAGAAATTGACCGCGCTAAGGCCCGTAAAGAACGTCACGAGGGGCGCTTAGAGGCACGCAGATTCAAACGTCGCAAACGCAAGGCAAGTAAAGCACACAAAGATAAGCGCGAAAGAATGCTTGCTGCGTGGCGATGGGCTGAACGTCAAGAACGGCATAAcc                                                                                                                               >  1:175293‑M2/126‑301 (MQ=255)`  
                                                                                                                                                                                `gtctgatgcctgacaccaaggacgtaatccgcatcctgcaagcgcgtatctatgagcaattaacgattgacctctgggaagacgcagaagacttgctcaatgaatacttggaggaagtcgaggaAGTCGCACGACAGAAAGAAATTGACCGCGCTAAGGCCCGTAAAGAACGTCACGAGGGGCGCTTAGAGGCACGCAGATTCAAACGTCGCAACCGCAAGGCACGTAAAGCACACAAAGCTAAGCGCGAAAGAATGCTTGCTGCGTGGCGATGGGCTGAACGTCAAGAACGGCGTAACCa                                                                                                                              <  1:82614‑M2/177‑1 (MQ=255)`  
                                                                                                                                                                                     `gagatgtgtataagagacagaatccgcatcctgcaagcgcgtatctatgagcaattaacgattgacctctgggaagacgcagaagacttgctcaatgaatacttggaggaagtcgaggaAGTCGCACGACAGAAAGAAATTGACCGCGCTAAGGCCCGTAAAGAACGTCACGAGGGGCGCTTAGAGGCACGCAGATTCAAACGTCGCAACCGCAAGGCACGTAAAGCACACAAAGCTAAGCGCGAAAGAATGCTTGCTGCGTGGCGATGGGCTGAACGTCAAGAACGGCGTAACCATGAgg                                                                                                                         <  1:117981‑M2/182‑1 (MQ=255)`  
                                                                                                                                                                                     `cgtgtgctcggagatgtgtataagagacagctgcaagcgcgtatctatgagcaattaacgattgacctctgggaagacgcagaagacttgctcaatgaatacttggaggaagtcgaggaAGTCGCACGACAGAAAGAAATTGACCGCGCTAAGGCCCGTAAAGAACGTCACGAGGGGCGCTTAGAGGCACGCAGATTCAAACGTCGCAACCGCAAGGCACGTAAAGCACACAAAGCTAAGCGCGAAAGAATGCTTGCTGCGTGGCGATGGGCTGAACGTCAAGAACGGCGTAACCATGAgg                                                                                                                         <  1:180101‑M2/182‑1 (MQ=255)`  
                                                                                                                                                                                     `atgcctgacaccaaggacgtaatccgcatcctgcaagcgcgtatctatgagcaattaacgattgacctctgggaagacgcagaagacttgctcaatgaatacttggaggaagtcgaggaAGTCGCACGACAGAAAGAAATTGACCGCGCTAAGGCCCGTAAAGAACGTCACGAGGGGCGCTTAGAGGCACGCAGATTCAAACGTCGCAACCGCAAGGCACGTAAAGCACACAAAGCTAAGCGCGAAAGAATGCTTGCTGCGTGGCGATGGGCTGAACGTCAAGAACGGCGTAACCATGAgg                                                                                                                         <  1:344497‑M2/182‑1 (MQ=255)`  
                                                                                                                                                                                     `aggcctgacaccaaggacgtaatccgcatcctgcaagcgcgtatctatgagcaattaacgattgacctctgggaagacgcagaagacttgctcaatgaatacttggaggaagtcgaggaAGTCGAACGACAGAAAGAAATTGACCGCGCTAAGGCCCGTAAAGAACGTCACGAGGGGCGCTTAGAGGCACGCAGATTCAAACGTCGCAACCGCAAGGCACGTAAAGCACACAAAGCTAAGCGCGAAAGAATGCTTGCTGCGTGGCGATGGGCTGAACGTCAAGAACGGCGTAACCATGAgg                                                                                                                         <  1:187349‑M2/182‑1 (MQ=255)`  
                                                                                                                                                                                        `cctgacaccaaggacgtaatccgcatcctgcaagcgcgtatctatgagcaattaacgattgacctctgggaagacgcagaagacttgctcaatgaatacttggaggaagtcgaggaAGTCGCACGACAGAAAGAAATTGACCGCGCTAAGGCCCGTAAAGAACGTCACGAGGGGCGCTTAGAGGCACGCAGATTCAAACGTCGCAACCGCAAGGCACGTAAAGCACACAAAGCTAAGCGCGACAGAATGCTTGCTGCGTGGCGATGGGCTGAACGGCAAGAACGgaggaaccactgtctct                                                                                                                      >  1:46054‑M2/117‑293 (MQ=255)`  
                                                                                                                                                                                           `gacaccaaggacgtaatccgcagcctgcaagcgcgtatctatgagcaattaacgattgacctctgggaagacgcagaagacttgctcaatgaatacttggaggaagtcgaggaAGTCGCACGAAAGAAAGAAATTGACCGCGCTAAGGCCCGTAAAGAACGTCACGAGGGGCGCTTAGAGGCACGCAGATTCAAACGTCGCAACCGCAAGGCACGTAAAGCACACAAAGCTAAGCGCGAAAGAATGCTTGCTGCGTGGCGATGGGCTGAACGTCAAGAACGGCGTAACCATGAGGTAGCTg                                                                                                                   <  1:212947‑M2/188‑1 (MQ=255)`  
                                                                                                                                                                                               `ccaaggacgtaatccgcatcctgcaagcgcgtatctatgagcaattaacgattgacctctgggaagacgcagaagacttgctcaatgaatacttggaggaagtcgaggaAGTCGCACGACAGAAAGAAATTGACCGCGCTAAGGCCCGTAAAGAACGTCACGAGGGGCGCTTAGAGGCACGCAGATTCAAACGTCGCAACCGCAAGGCACGTAAAGCACACAAAGCTAAGCGCGAAAGAATGCTTGCTGCGTGGCGATGGGCTGAACGTCAAGAACGGCGTAACCATGAGGTAGctgtctg                                                                                                               >  1:327488‑M2/110‑298 (MQ=255)`  
                                                                                                                                                                                               `ccaaggacgtaatccgcatcctgcaagcgcgtatctatgagcaattaacgattgacctctgggaagacgcagaagacttgctcaatgaatacttggaggaagtcgaggaAGTCGCACGACAGAAAGAAATTGACCGCGCTAAGGCCCGTAAAGAACGTCACGAGGGGCGCTTAGAGGCACGCAGATTCAAACGTCGCAACCGCAAGGCACGTAAAGCACACAAAGCTAAGCGCGAAAGAATGCTTGCTGCGTGGCGATGGGCTGAACGTCAAGAACGGCGTAAACATGAGGTAGCTGTAGa                                                                                                               >  1:23391‑M2/110‑301 (MQ=255)`  
                                                                                                                                                                                                `caaggacgtaatccgcatcctgcaagcgcgtatctatgagcaattaacgattgacctctgggaagacgcagaagacttgctcaatgaatacttggaggaagtcgaggaAGTCGCACGACAGAAAGAAATTGACCGCGCTAAGGCCCGTAAAGAACGTCACGAGGGGCGCTTAGAGGCACGCAGATTCAAACGTCGCAACCGCAAGGCACGTAAAGCACACAAAGCTAAGCGCGAAAGAATGCTTGCTGCGTGGCGATGGGCTGAACGTCAAGAACGGCGTAACCATGAGGTAgctgtctgt                                                                                                              >  1:49264‑M2/109‑297 (MQ=255)`  
                                                                                                                                                                                                `aaaggacgtaatccgaatccttcaagcgcgtatctatgagcaattaacgattgacctctgggaagacgcagaagacttgctaaatgaatacttggaggaagtcgaggaAGTCGCACGACAGAAAGAAATTGACCGCGCTAAGGCCCGTAAAGAACGTCACGAGGGGCGCTTAGAGGCACGCAGATTCAAACGTCGCAACCGCAAGGCACGTAAAGCACACAAAGCTAAGCGCGAAAGAATGCTTGCTGCGTGGCGATGGGCTGAACGTCAAGAACGGCGTAACCATGAGGTAGCTGTAGAt                                                                                                              <  1:72146‑M2/193‑1 (MQ=255)`  
                                                                                                                                                                                                    `gaggtaatccgaatcatgaaatcgcgtatcaatgagtaattaacgattgacatctgggaaggcgcagaagacttgatcaaggaatacttggaggaagtcgaggaAGTCGCACGACAGAAAGAAATTGACCGCGCTAAGGCCAGGAAAGAACGTCACGAGGGGCGCTTAGAGGCACGCAGATTAAAACGTCGCAACCGCAAGGCACGTAAAGCACACAAAGCTAAGCGCGAAAGAATGCTTGCTGCGTGGCGATGGGCTGAACGTCAAGAACGGCGTAACCATGAGGTAGCTGTAGATGTAc                                                                                                          <  1:85786‑M2/197‑1 (MQ=255)`  
                                                                                                                                                                                                    `gacgtaatccgcatcctgcaagcgcgaatcgatgaacaattaacgattgacctctggaaagacgcagaagacttgcgcaatgaatacttggaggaagtcgaggaAGTCGCACGACAGAAAGAAATTGACCGCGCTAAGGCCCGTAAAGAACGTCACGAGGGGCGCTTATAGGCACGCAGATTCAAACGTCGCAACCGCAAGGCACGTAAAGCACACAAAGCTAAGCGCGAAAGAATGCTTGCTGCGTGGCGATGGGCTGAACGTCAAGAACGGCGTAACCATGAGGTAGCTGTAGATGTAc                                                                                                          <  1:100161‑M2/197‑1 (MQ=255)`  
                                                                                                                                                                                                     `acgtaatccgcatcctgcaagcgcgtatctatgagcaattaacgattgacctctgggaagacgcagaagacttgctcaatgaatacttggaggaagtcgaggaAGTCGCACGACAGAAAGAAATTGACCGCGCTAAGGCCCGTAAAGAACGTCACGAGGGGCGCTTAGAGGCACGCAGATTCAAACGTCGCAACCGCAAGGCACGTAAAGCACACAAAGCTAAGCGCGAAAGAATGCTTGCTGCGTGGCGATGGGCTGAACGTCAAGAACGGCGTAACCATGAGGTAGCTGTAGATGTACt                                                                                                         >  1:78731‑M2/104‑301 (MQ=255)`  
                                                                                                                                                                                                      `cgtaatccgcatcctgcaagcgcgtatctatgagcaattaacgattgacctctgggaagacgcagaagacttgctcaattaatacttggaggaagtcgaggaAGTCGCACGACAGAAAGAAATTGACCGCGCTAAGGCCCGTAAAGAACGTCACGAGGGGCGCTTAGAGGCACGCAGATTCAAACGTCGCAACCGCAAGGCACGTAAAGCACACAAAGCTAAGCGCGAAAGAATGCTTGCTGCGTGGCGATGGGCTGAACGTCAAGAACGGCGTAACCATGAGGTAGCtgtagatctgtct                                                                                                        >  1:3373‑M2/103‑295 (MQ=255)`  
                                                                                                                                                                                                      `cgtaatccgcatcctgcaagcgcgtatctatgagcaattaacgattgacctctgggaagacgcagaagacttgctcaatgaatacttggaggaagtcgaggaAGTCGCACGACAGAAAGAAATTGACCGCGCTAAGGCCCGTAAAGAACGTCACGAGGGGCGCTTAGAGGCACGCAGATTCAAACGTCGCAACCGCAAGGCACGTAAAGCACACAAAGCTAAGCGCGAAAGAATGCTTGCTGCGTGGCGATGGGCTGAACGTCAAGAACGGCGTaaccatgaggtagcctgtctcttatac                                                                                                        >  1:290166‑M2/103‑288 (MQ=255)`  
                                                                                                                                                                                                      `caccaaggacgtaatccgcatcctgcaagcgcgtatctatgagcaattaacgattgacctctgggaagacgcagaagacttgctcaatgaatacttggaggaAGTCGCACGACAGAAAGAAATTGACCGCGCTAAGGCCCGTAAAGAACGTCACGAGGGGCGCTTAGAGGCACGCAGATTCAAACGTCGCAACCGCAAGGCACGTAAAGCACACAAAGCTAAGCGCGAAAGAATGCTTGCTGCGTGGCGATGGGCTgaacgtcaagaacggcgtaaccactgtctcttatacacatctccg                                                                                                        >  1:154809‑M2/103‑279 (MQ=255)`  
                                                                                                                                                                                                         `tgtgtataagagacaggcgcgtatctatgagcaattaacgattgacctctgggaagacgcagaagacttgctcaatgaatacttggaggaagtcgaggaAGTCGCACGACAGAAAGAAATTGACCGCGCTAAGGCCCGTAAAGAACGTCACGAGGGGCGCTTAGAGGCACGCAGATTCAAACGTCGCAACCGCAAGGCACGTAAAGCACACAAAGCTAAGCGCGAAAGAATGCTTGCTGCGTGGCGATGGGCTGAACGTCAAGAACGGCGTAACCATGAGGTAGCTGTAGATGTACTAGga                                                                                                     <  1:128145‑M2/202‑1 (MQ=255)`  
                                                                                                                                                                                                         `aatccgcatcctgcaagcgcgtatctatgagcaattaacgattgacctctgggaagacgcagaagacttgctcaatgaatacttggaggaagtcgaggaAGTCGCACGACAGAAAGAAATTGACCGCGCTAAGGCCCGTAAAGAACGTCACGAGGGGCGCTTAGAGGCACGCAGATTCAAACGTCGCAACCGCAAGGCACGTAAAGCACACAAAGCTAAGCGCGAAAGAATGCTTGCTGCGTGGCGATGGGCTGAACGTCAAGAACGGCGTAACCATGAGGTAGCTGTAGATGTACTAGga                                                                                                     <  1:167261‑M2/202‑1 (MQ=255)`  
                                                                                                                                                                                                         `aatccgcatcctgcaagcgcgtatctatgagcaattaacgattgacctctgggaagacgcagaagacttgctcaatgaatacttggaggaagtcgaggaAGTCGCACGACAGAAAGAAATTGACCGCGCTAAGGCCCGTAAAGAACGTCACGAGGGGCGCTTAGAGGCACGCAGATTCAAACGTCGCAACCGCAAGGCACGTAAAGCACACAAAGCTAAGCGCGAAAGAATGCTTGCTGCGTGGCGATGGGCTGAACGTCAAGAACGGCGTAACCATGAGGTAGCTGTAGATGTACTAGga                                                                                                     >  1:60626‑M2/100‑301 (MQ=255)`  
                                                                                                                                                                                                         `aatacgaatcctgcaagcgcgtatctatgagcaattaacgattgacctctgggaagacgcagaagacttgctcaatgaatacttggaggaagtcgaggaAGTCGCACGACAGAAAGAAATTGACCGCGCTAAGGCCCGTAAAGAACGTCACGAGGGGCGCTTAGAGGCACGCAGATTCAAACGTCGCAACCGCAAGGCACGTAAAGCACACAAAGCTAAGCGCGAAAGAATGCTTGCTGCGTGGCGATGGGCTGAACGTCAAGAACGGCGTAACCATGAGGTAGCTGTAGATGTACTAGga                                                                                                     <  1:223846‑M2/202‑1 (MQ=255)`  
                                                                                                                                                                                                                   `ctgcaagcgcgtatctatgagcaattaacgattgacctctgggaagacgcagaagacttgctcaatgaatacttggaggaagtcgaggaAGTCGCACGACAGAAAGAAATTGACCGCGCTAAGGCCCGTAAAGAACGTCACGAGGGGCGCTTAGAGGCACGCAGATTCAAACGTCGCAACCGCAAGGCACGTAAAGCACACAAAGCTAAGCGCGAAAGAATGCTTGCTGCGTGGCGATGGGCtgaacgtcaagaacggcgtaaccatgaggtctgtctcttatacacatctccgagcccac                                                                                           >  1:39988‑M2/90‑272 (MQ=255)`  
                                                                                                                                                                                                                      `aaagggaaagtattgaagaaattaacgattgacctctgggaaacgccagaggattggctaaataatacttggaggaaggtcgaggaGGTAAAACGAC‑GGAAGAAAATTACCGGGGCAAGGCCCGTAAAGAACGTCACGAGGGGCGCTTAGAGGCACGCAGATTCAAACGTCGCAACCGCAAGGCACGTAAAGCACACAAAGCTAAGCGCGAAAGAATGCTTGCTGCGTGGCGATGGGCTGAACGTCAAGAACGGCGTAACCATGAGGTAGCTGTAGATGTACTAGGAAGAACCAATAACGc                                                                                       <  1:54240‑M2/215‑1 (MQ=255)`  
                                                                                                                                                                                                                            `ggaatctatgagcaattaacgattgacctctgggaagacgcagaagacttgctcaatgaatacttggaggaagtcgaggaAGTCGCACGACAGAAAGAAATTGACCGCGCTAAGGCCCGTAAAGAACGTCACGAGGGGCGCTTAGAGGCACGCAGATTCAAACGTCGCAACCGCAAGGCACGTAAAGCACACAAAGCTAAGCGCGAAAGAATGCTTGCTGCGTGGCGATGGGCTGAACGTCAAGAACGGCGTAACCATGAGGTAGCTGTAGATGTACTAGGAAGAACCAATAACGCTATGc                                                                                  <  1:6357‑M2/221‑1 (MQ=255)`  
                                                                                                                                                                                                                             `tgcaagcgcgtatctatgagcaattaacgattgacctctgggaagacgcagaagacttgctcaatgaatacttggaggaAGTCGCACGACAGAAAGAAATTGACCGCGCTAAGGCCCGTAAAGAACGTCACGAGGGGCGCTTAGAGGCACGCAGATTCAAACGTCGCAACCGCAAGGCACGTAAAGCACACAAAGCTAAGCGCGAAAGAATGCTTGCTGCGTGGCGATGGGCTGAACGTCAAGAACGGCGTAACCATGAGGTAGCTGTAGATGTACTAGGAAGAACCAATAACGCTATGct                                                                                 >  1:279002‑M2/80‑301 (MQ=255)`  
                                                                                                                                                                                                                              `tatctatgagcaattaacgattgacctctgggaagacgcagaagacttgctcaatgaatacttggaggaagtcgaggaAGTCGCACGACAGAAAGAAATTGACCGCGCTAAGGCCCGTAAAGAACGTCACGAGGGGCGCTTAGAGGCACGCAGATTCAAACGTCGCAACCGCAAGGCACGTAAAGCACACAAAACTAAGCGCGAAAGAATGCTTGCTGCGTGGCGATGGGCTGAACGTCAAGACCGGCGTAACCATGAGGTAGCTGTAGAAGAACTAGGCAGAACCAATAACGCTATGctc                                                                                >  1:306479‑M2/79‑301 (MQ=255)`  
                                                                                                                                                                                                                                 `ttatgagaaattaacgattgacctctgggaagacgcagaagactggctcaatgaatacttggagcaagtcgaggaAGAAGCACGACAGAAAGAAATTGACCGCGCTAAGGCCCGTAAAGAACGTCACGAGGGGCGCTTAGAGGCACGCAGATTCAAACGTCGAAACCGCAAGGCACGTAAAGCACACAAAGCTAAGCGCGAAAGAATGCTTGCTGCGTGGCGATGGGCTGAACGTCAAGAACGGCGTAACCATGAGGTAGCTGTAGATGTACTAGTAAGAACCAATAACGCTATGCTCTgg                                                                             <  1:135562‑M2/226‑1 (MQ=255)`  
                                                                                                                                                                                                                                 `agcgcgtatctatgagcaattaacgattgacctctgggaagacgcagaagacttgctcaatgaatacttggaggaAGTCGCACGACAGAAAGAAATTGACCGCGCTAAGGCCCGTAAAGAACGTCACGAGGGGCGCTTAGAGGCACGCAGATTCAAACGTCGCAACCGCAAGGCACGTAAAGCACACAAAGCTAAGCGCGAAAGAATGCTTGCTTCGTGGCGATGGGCTGAACGTCAAGAACggcgtaaccatgaggtagctgtagatgtacagctacctcatggttacgccattcttgac                                                                             <  1:25869‑M2/226‑30 (MQ=255)`  
                                                                                                                                                                                                                                    `agagcaattaacgatggacctctgggaagacgcagaagacttgctcaaggaatacttggaggaagtcgaggaAGTAGCACGACAGAAAGAAATTGCCCGCGCTAAGGCCCGTAAAGAACGTCACGAGGGGCGCTTAGAGGCACGCAGATTCAAACGTCGCAACCGCAAGGCACGTAAAGCACACAAAGCTAAGCGCGAAAGAATGCTTGCTGCGTGGCGATGGGCTGAACGTCAAGAACGGCGTAACCATGAGGTAGCTGTAGATGTACTAGGAAGAACCAATAACGCTATGCTCTGGGTc                                                                          <  1:70175‑M2/229‑1 (MQ=255)`  
                                                                                                                                                                                                                                        `caattaacgattgacctctgggaagacgcagaagacttgctcaatgaatacttggaggaagtcgaggaAGTCGCACGACAGAAAGAAATTGACCGCGCTAAGGCCCGTAAAGAACGTCACGAGGGGCGCTTAGAGGCACGCAGATTCAAACGTCGCAACCGCAAGGCACGTAAAGCACACAAAGCTAAGCGCGAAAGAATGCTTGCTGCGTGGCGATGGGCTGAACGTCAAGAACGGCGTAACCATGAGGTAGCTGTAGATGTACTAGGAAGAACCAATAACGCtatgctctgtctcttat                                                                      >  1:189826‑M2/69‑293 (MQ=255)`  
                                                                                                                                                                                                                                        `caattaacgattgacctctgggaagacgcagaagacttgctcaatgaatacttggaggaagtcgaggaAGTCGCACGACAGAAAGAAATTGACCGCGCTAAGGCCCGTAAAGAACGTCACGAGGGGCGCTTAGAGGCACGCAGATTCAAACGTCGCAACCGCAAGGCACGTAAAGCACACAAAGCTAAGCGCGAAAGAATGCTTGCTGCGTGGCGATGGGCTGAACGTCAAGAACGGCGTAACCATGAGGTAGCTGTAGATGTACTAGGAAGAACCAATAACGCCATGCCCTGGGTCAACa                                                                      >  1:65740‑M2/69‑301 (MQ=255)`  
                                                                                                                                                                                                                                        `aaattaacgattgacctccgggaaaacgcagaagacttgctcaatgaatacttggaggaaggcgaggaAGTCGCAGGACAGAAAGAAATTGACCGCGCTAAGGCCCGTAAAGAACGTCACGAGGGGCGCTTAGAGGCACGCAGATTCAAACGTCGCAACCGCAAGGCACGTAAAGCACACAAAGCTAAGCGCGAAAGAATGCTTGCTGCGTGGCGATGGGCTGAACGTCAAGAACGGCGTAACCATGAGGTAGCTGTAGATGTACTAGGAAGAACCAATAACGCTATGCTCTGGGTCAACa                                                                      <  1:321050‑M2/233‑1 (MQ=255)`  
                                                                                                                                                                                                                                                     `taacgattgacccctgggaaggcgcagaaagctggctcaattaatacgtggaggaAGTCGCACGACAGAAAGAAATTGACCGCGCTAAGGCCCGTAAAGAACGTCACGAGGGGCGCTTAGAGGCACGCAGATTCAAACGTCGCAACCGCAAGGCACGTAAAGCACACAAAGCTAAGCGCGAAAGAATGCTTGCTGCGTGGCGATGGGCTGAACGTCAAGAACGGCGTAACCATGAGGTAGCTGTAGATGTACTAGGAAGAACCAATAACGCTATGCTCTGGGTCAACATGTTCTCTGGGGa                                                         <  1:296846‑M2/246‑1 (MQ=255)`  
                                                                                                                                                                                                                                                      `cctctgggaagacgcagaagacttgctcaatgaatacttggaggaagtcgaggaAGTCGCACGACAGAAAGAAATTGACCGCGCTAAGGCCCGTAAAGAACGTCACGAGGGGCGCTTAGAGGCACGCAGATTCAAACGTCGCAACCGCAAGGCACGTAAAGCACACAAAGCTAAGCGCGAAAGAATGCTTGCTGCGTGGCGATGGGCTGAACGTCAAGAACGGCGTAACCATGAGGTAGCTGTAGATGTACTAGGAAGAACCAATAACGCTATGCTCAGGGTCAACATGTTCTCTGGGGAc                                                        >  1:43217‑M2/55‑301 (MQ=255)`  
                                                                                                                                                                                                                                                      `cctctgggaagacgcagaagacttgctcaatgaatacttggaggaagtcgaggaAGTCGCACGACAGAAAGAAATTGACCGCGCTAAGGCCCGTAAAGAACGTCACGAGGGGCGCTTAGAGGCACGCAGATTCAAACGTCGCAACCGCAAGGCACGTAAAGCACACAAAGCTAAGCGCGAAAGAATGCTTGCTGCGTGGCGATGGGCTGAACGTCAAGAACGGCGTAACCATGAGGTAGCTGTAGATGTACTAGGAAGAACCAATAACGCTATGATCTGGGTCAAAATGTTCTCTGGGGAc                                                        >  1:157305‑M2/55‑301 (MQ=255)`  
                                                                                                                                                                                                                                                      `cctctgggaagacgcagaagacttgctcaatgaatacatggaggaagtcgaggaAGTCGCACGAAAGAAAGAAATTGACCGCGCTAAGGCCCGTAAAGAAAGTACCGAGGGGCGCTTAGAGGCCCGCAGATTCAAACGTCGCAACCGCAAGGCACGTAAAGCACACAAAGCTAAGCGCGAAAGAATGCTTGCTGCGTGGCGATGGGCTGGACGTCAAGAACGGCGTAACGATGAGGTAGATGTAGCTGTAATAGGAAGAACCAATAACGACATGCTCTGGGTCTACCTATTATcttggggg                                                        >  1:92797‑M2/55‑299 (MQ=255)`  
                                                                                                                                                                                                                                                              `aagacgaagaagactggcacaatgaataattggaggaaggcgaggaAGTCGCAAGAAAGAAAGAAAATGACCGCGCTAAGGCCCGTAAAGAACGTCACGAGGGGCGCTTAGAGGCACGCAGATTCAAACGTCGCAACCGAAAGGCACGTAAAGCACACAAAGCTAAGCGCGAAAGAATGCGTGCTGCGTGGCGATGGGCTGAACGTCAAGAAAGGCGTAACCATGAGGTAGCTGTAGATGTACTAGGAAGAACCAATAACGCTATGCTCTGGGTCAACATGTTCTCTGGGGACTTTAAGgc                                                <  1:57114‑M2/255‑1 (MQ=255)`  
                                                                                                                                                                                                                                                                      `gaagacttgctcaatgaatacttggaggaagtcgaggaAGTCGCACGACAGAAAGAAATTGACCGCGCTAAGGCCCGTAAAGAACGTCACGAGGGGCGCTTAGAGGCACGCAGATTCAAACGTCGCAACCGCAAGGCACGTAAAGCACACAAAGCTAAGCGCGAAAGAATGCTTGCTGCGTGGCGATGGGCTGAACGTCAAGAACGGAGTAACCATGAGGTAGCTGTAGATGTACTAGGAAGAACCAATAACGCTATGCTCTGGGTCAACATGTTCTCTGGGGACTTTAAGGCGCTTGAgg                                        <  1:114535‑M2/263‑1 (MQ=255)`  
                                                                                                                                                                                                                                                                        `gtgtataagagacaagtacttggaggaagtcgaggaAGTCGAACGACAGAAAGAAATTGACCGCGCTAAGGCCCGTAAAGAACGTCACGAGGGGCGCTTAGAGGCACGCAGATTCAAACGTCGCAACCGCAAGGCACGTAAAGCACACAAAGCTAAGCGCGAAAGAATGCTTGCTGCGTGGCGATGGGCTGAACGTCAAGGACGGCGTAACCATGAGGTAGCTGTAGATGTACTAGGAAGAACCAATAACGCTATGCTCTGGGTCAACATGTTCTCTGGGGACTTTAAGGCGCTTGAGGaa                                      <  1:151302‑M2/265‑1 (MQ=255)`  
                                                                                                                                                                                                                                                                         `gacttgctcaatgaatacttggaggaagtcgaggaAGTCGCACGACAGAAAGAAATTGACCTCGCTAAGGCCCGTAAAGAACGTCACGAGGGGCGCTTAGAGGCACGCAGATTCAAACGTCGCAACCGCAAGGCACGTAAAGCACACAAAGCTAAGCGCGAAAGAATGCTTGCTTCGTGGCGATGGGCTGAACGTCAAGAACGGCGTAACCATGAGGTAGCTGTCGATGTACTAGGAAGAAACAATAACGCTATGCTCTGGGTCAACATGTTATCTGGGGACTTTAGGGCGCTTGAGGAAc                                     >  1:335124‑M2/36‑301 (MQ=255)`  
                                                                                                                                                                                                                                                                                       `atacttggaggaagtcgaggaAGTCGCACGACAGAAAGAAATTGACCGCGCTAAGGCCCGTAAAGAACGTCACGAGGGGCGCTTAGAGGCACGCAGATTCAAACGTCGCAACCGCAAGGCACGTAAAGCACACAAAGCTAAGCGCGAAAGAATGCTTGCTGCGTGGCGATGGGCTGAACGTCAAGAACGGCGTAACCATGAGGTAGCTGTAGATGTACTAGGAAGAACCAATAACGCTATGCTCTGGGTCAACATGTTCTCTGGGGACTTTAAGGCGCTTGAGGAACGAATCGCGCTGCAc                       <  1:272481‑M2/280‑1 (MQ=255)`  
                                                                                                                                                                                                                                                                                       `agacttggaggaagtagaggaAGTCGCACGACAGAAAGAAATTGACCGCGCTAAGGCCCGTAAAGAACGTCACGAGGGGCGCTTAGAGGCACGCAGATTCAAACGTCGCAACCGCAAGGCACGTAAAGCACACAAAGCTAAGCGCGAAAGAATGCTTGCTGCGTGGCGATGGGCTGAACGTCAAGAACGGCGTAACCATGAGGTAGCTGTAGATGTACTAGGAAGAACCAATAACGCTATGCTCTGGGTCAACATGTTCTCTGGGGACTTTAAGGCGCTTGAGGAACGAATCGCGCTGCAc                       <  1:17131‑M2/280‑1 (MQ=255)`  
                                                                                                                                                                                                                                                                                        `tacttggaggaagtcgaggaAGTCGCACGACAGAAAGAAATTGACCGCGCTAAGGCCCGTAAAGAACGTCACGAGGGGCGCTTAGAGGCACGCCGATTCAAACGTCGCAACCGCAAGTCACGTAAAGCACACAAAGCTAAGCGCGAACGAATGCTTGCTGCGTGGCGATGGGCTGAACGTCAAGAACGGCGTAACCATGAGGTAGCTGTAGATGTACTAGGAAGAACCAATACCGCTCTGCTCTGGGTCAACATGTTCTCTGGggactttaaggcgcttgaggctgtctcttatacacatt                      >  1:220427‑M2/21‑283 (MQ=255)`  
                                                                                                                                                                                                                                                                                         `acttggaggaagtcgaggaAGTCGCACTACAGAAAGAAATTGACCGCGCTAAGGCCCGTAAAGAACGTCACGAGGGGCGCTTAGAGGCACGCAGATTCAAACGTCGCAACCGCAAGGCAAGTAAAGCACACAAAGCTAAGCTCGAAAGAATGCTTGCTGCGTGGCGATGGGCTGCATGTCAAGAACGGCGTAACCATGAGGTAGCTGTAGATGTACTAGGAAGAACCAATAACGCTATGCTCTGGGTCAACATGATCTCTGGGGGCTTTAAGGCGCTTGAGGAACGAATCGAgctgcccct                     >  1:339718‑M2/20‑297 (MQ=255)`  
                                                                                                                                                                                                                                                                                         `acttggaggaagtcgaggaAGTCGCACGACAGAAAGAAATTGACCGCGCTAAGGCCCGTAAAGAACGTCACGAGGGGCGCTTAGAGGCACGCAGATTCAAACGTCGCAACCGCAAGGCACGTAAAGCACACAAAGCTAAGCGCGAAAGAATGCTTGCTGCGTGGCGATTGGCTGAACGTCAAGAACGGCGTAACCATGAGGTAGCTGTAGATGTACTAGGAAGAACCAATAACGCTATGCTCTGGGTCAACATGTTCTCTGGGGACTTTAAGGCGCTTGAGGAACGAATCGCGCTACACTg                     >  1:318362‑M2/20‑301 (MQ=255)`  
                                                                                                                                                                                                                                                                                            `gggaggaagtcggtgaAGGCGCAAGACAAAAAGAAATTGACCGCGCTAAGGCCCGTAAAGAACGTCACGAGGGGCGCTTAGAGGCACGCAGATTCAAACGTCGCAACCGCAAGGCACGTAAAGCACACAAAGCTAAGCGCGAAAGAATGCTTGCTGCGTGGCGATGGGCTGAACGTCAAGAACGGCGTAACCATGAGGTAGCTGTAGATGTACTAGGAAGAACCAATAACGCTATGCTCTGGGTCAACATGTTCTCTGGGGACTTTAAGGCGCTTGAGGAACGAATCGCGCTGCACTGGCg                  <  1:121723‑M2/285‑1 (MQ=255)`  
                                                                                                                                                                                                                                                                                                    `gtcgaggaAGTCGCACGACAGAAAGAAATTGACCGCGCTAAGGCCCATAAAGAACGTCACGAGGGGCGCTTAGAGGCACGCAGATTCAAACGTCGCAACCGCAAGGCACGTAAAGCACACAAAGCTAAGCGCGAAAGAATGCTTGCTGCGTGGCGATGGGCTGAACGTCAAGAACGGCGTAACCATGAGGTAGCTGTAGATGTACTAGGAAGAACCAATAACGCTATGCTCTGGGTCAACATGTTCTCTGGGGACTTTAAGGCGCTTGAGGAACGAATCGCGCTGCACTGGCGTAATGCTg          >  1:247712‑M2/9‑301 (MQ=255)`  
                                                                                                                                                                                                                                                                                                       `gaggaAGTCGCACGACAGAAAGAAATTGACCGCGCTAAGGCCCGTAAAGAACGTCACGAGGGGCGCTTAGAGGCACGCAGATTCAAACGTCGCAACCGCAAGGCACGTAAAGCACACAAAGCTAAGCGCGAAAGAATGCTTGCTGCGTGGCGATGGGCTGAACGTCAAGAACGGCGTAACCATGAGGTAGCTGTAGATGTACTAGGAAGAACCAATAACGCTATGCTCTGGGTCAACATGTTCTCTGGGGACTTTAAGGCGCTTGAGGAATGAATCGCGCTGCACTGGCGTAATGCTGAcc       <  1:3637‑M2/296‑1 (MQ=255)`  

                                                                                                                                                                                                                                                                                                            `|`                                                                                                                                                                                                                                                                                                              
`GGATTGCGAGCGTTTCAACAATGATGCCCATTATAAATACGCTGAGATTGCAAGCGACATCATTGATTGCAATTCGGATGAGCATGATGAGTTAACTGGATGGGATGGTGAGTTTGTTGAAACTTGTAAACTAATCCGCAAGTTCTTTGAGGGCATCGCCTCATTCGACATGCATAGCGGGAACATCATGTTCTCAAATGGAGACGTACCATACATCACCGACCCGGTATCATTCTCGCAGAAGAAAGACGGTGGCGCATTCAGCATCGACCCTGAGGAACTCATCAAGGAAGTCGAGGAAGTCGCACGACAGAAAGAAATTGACCGCGCTAAGGCCCGTAAAGAACGTCACGAGGGGCGCTTAGAGGCACGCAGATTCAAACGTCGCAACCGCAAGGCACGTAAAGCACACAAAGCTAAGCGCGAAAGAATGCTTGCTGCGTGGCGATGGGCTGAACGTCAAGAACGGCGTAACCATGAGGTAGCTGTAGATGTACTAGGAAGAACCAATAACGCTATGCTCTGGGTCAACATGTTCTCTGGGGACTTTAAGGCGCTTGAGGAACGAATCGCGCTGCACTGGCGTAATGCTGACCGGATG  >  NC_001604.1/2437‑3037`

  

Alignment Legend

Aligned base mismatch/match (shaded by quality score): `ATCG/ATCG` `< 3 ≤` `ATCG/ATCG` `< 10 ≤` `ATCG/ATCG` `< 22 ≤` `ATCG/ATCG` `< 34 ≤` `ATCG/ATCG` `< 38 ≤` `ATCG/ATCG`

Unaligned base: `atcg`    Masked matching base: `atcg`    Alignment gap: `‑`    Deleted base: `‑`

Reads not counted as support for junction

read\_name Not counted due to insufficient overlap past the breakpoint.

read\_name Not counted due to not crossing MOB target site duplication.
